# Evolutionary Stability in the Asymmetric Volunteer's Dilemma

**DOI:** 10.1371/journal.pone.0103931

**Published:** 2014-08-11

**Authors:** Jun-Zhou He, Rui-Wu Wang, Yao-Tang Li

**Affiliations:** 1 Statistics and Mathematics College, Yunnan University of Finance and Economics, Kunming, Yunnan, China; 2 State Key Laboratory of Genetic Resources and Evolution, Kunming Institute of Zoology, Chinese Academy of Science, Kunming, Yunnan, China; 3 School of Mathematics and Statistics, Yunnan University, Kunming, Yunnan, China; Hong Kong Baptist University, China

## Abstract

It is often assumed that in public goods games, contributors are either strong or weak players and each individual has an equal probability of exhibiting cooperation. It is difficult to explain why the public good is produced by strong individuals in some cooperation systems, and by weak individuals in others. Viewing the asymmetric volunteer's dilemma game as an evolutionary game, we find that whether the strong or the weak players produce the public good depends on the initial condition (i.e., phenotype or initial strategy of individuals). These different evolutionarily stable strategies (ESS) associated with different initial conditions, can be interpreted as the production modes of public goods of different cooperation systems. A further analysis revealed that the strong player adopts a pure strategy but mixed strategies for the weak players to produce the public good, and that the probability of volunteering by weak players decreases with increasing group size or decreasing cost-benefit ratio. Our model shows that the defection probability of a “strong” player is greater than the “weak” players in the model of Diekmann (1993). This contradicts Selten's (1980) model that public goods can only be produced by a strong player, is not an evolutionarily stable strategy, and will therefore disappear over evolutionary time. Our public good model with ESS has thus extended previous interpretations that the public good can only be produced by strong players in an asymmetric game.

## Introduction

In social groups, voluntary acts play a critical role in the maintenance and evolution of cooperation [1]–[5]. Volunteers provide public goods for the group by contributing services or by punishing cheaters. For example, in meerkats watchful sentinels might warn other individuals of predators by using alarm calls [6], [7], or a commercial company might sanction another that is violating the price level agreed upon by the member firms of a cartel [8]. However, potential volunteers face a dilemma in that volunteers produce the public good at a cost to themselves, whereas cheaters exploit the benefits but forego the costs [9]–[14]. The resulting question then arises: which individuals pay the extra cost to voluntarily produce a public good?

The volunteer's dilemma (VD) is an *N*-person, binary-choice game designed to explain why the participants would be inclined to pay the cost of providing a public good shared by every individual in a social group [7], [15]–[18]. In this game, each individual faces a binary set of options, including a costly to volunteer and a costless no volunteer choice, and the symmetric Nash equilibrium that involves mixed strategies which imply each individual has equal probability to cooperate [15], [19]. The conventional form of the VD makes the basic assumption that partners interact symmetrically, with equal payoffs in a game of cooperative interaction [15]. However, almost all of the well-studied inter-specific cooperation systems [20]–[25], and intra-specific systems [26]–[29], have shown that cooperative individuals interact asymmetrically. This asymmetric interaction could, for example, be a difference in resource allocation among players, a difference in the probability of winning a fight with others, or any other similar characteristics between dominant and subordinate individuals [30], [31]. Dominant and subordinate are terms in theoretical models more frequently referred to as “strong” or “weak” players, respectively [30]. This asymmetric interaction leads to different payoffs of the involved players and might therefore influence the choices of action of individuals [18], [24], [30]–[36].

Selten (1980) developed an asymmetry model that assumed that the distribution of the payoff is unequal between players [37]. Using evolutionary game theory with a two-person game, his model showed that the public good would only be produced by the “strong” player. However, these theoretical results were not easily reconciled with experimental observations, because the public good is produced by weak players in some systems [29]. The fact that the two-person game model loses generality might be what raises the difficulty of Selten's (1980) model in explaining why public goods are produced by the “weak” players in some cooperative systems.

Another asymmetric volunteer's dilemma game developed by Diekmann (1993), but with N-persons, introduced an unequal distribution of costs and interests among different players. Using standard game theory, Diekmann's model showed that players might adopt mixed strategies. The mixed strategies of the asymmetric game predicted that the players with lower costs (i.e., “strong” players) will contribute less frequently than players with high costs (i.e., “weak” players), a result contradicting many empirical observations (e.g., Harsanyi-Selten theory, Schelling's “prominence theory”; see [8], [37], [38]).

Clearly, the multiplayer assumption of Diekman's (1993) model is consistent with real cooperative systems, whilst the evolutionary game theory used by Selten (1980) is more universal. A combination of the advantages of both approaches may thus give further insight into the public goods dilemma.

In this paper, we follow Diekmann (1993) in enriching Selten's (1980) model to incorporate some important aspects of asymmetric systems, namely that the model should contain the *N*-person game, and both symmetric and asymmetric interactions. In our model, Diekmann's paradox disappears. We find the evolutionarily stable strategy (ESS) of the asymmetric volunteer's dilemma, and describe the conditions how the public good is produced by both “strong” and “weak” players in an asymmetric game.

## Methods

### Asymmetric Volunteer's Dilemma Game

Diekmann's (1993) asymmetric volunteer's dilemma game introduced an unequal distribution of costs 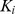 and interests 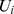 among 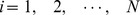 players, and analyzed the binary-decision *N*-Person game with each player *i*'s decision an alternative between 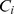 (cooperation) and 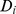 (defection). Assuming that for all *i*'s 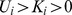, the payoff structure is as follows:

- Employing strategy 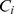 always yields the net payoff of 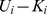; whereas
- Employing strategy 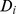 yields the maximum payoff of 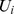 whenever at least one other player employs strategy 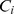 (“volunteering” for other players); otherwise
- If all players employ strategy 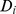, they yield a payoff of 0.

Based on this structure, the asymmetric volunteer's dilemma game has *N* efficient and strict equilibriums with exactly one “volunteer” and *N*-1 “free-riders”. Moreover, an additional equilibrium point in mixed strategies exists [8].

In real systems, such as bees, ants or monkeys, there is a single dominant reproductive individual. Therefore we will study a special case of the asymmetric volunteer's dilemma game with one “strong” player and *N-1* “weak” players. This implies that the “strong” player will obtain either greater utility 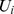 or a lower cost 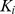 than “weak” players [8]. In addition, we assume that *N-1* “weak” players have an equal degree of weakness (i.e. *N-1* “weak” players possess an equal payoff) and the population of *N-1* “weak” players is monomorphic in which all “weak” players use the same strategy [39]. From the assumptions, we have:
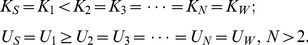
(2.1)Here 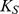/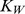 and 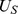/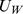 are the cost of cooperation and unity of public goods to the “strong/weak” player, respectively.

If we let 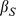 and 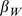 be the defection probabilities of the “strong” player and *N-1* “weak” co-players respectively, and let 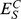,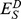 represent the payoff of the cooperation (C) and defection (D) strategies of “strong” players and a similar notation for 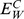,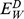 for the “weak” players, then:
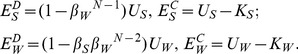
(2.2)Thus, the average payoffs for the “strong” and “weak” players respectively are:
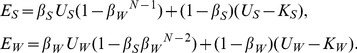
(2.3)
Equation (2.3) implies the payoffs of both “strong” and *N-1* “weak” players depend on the mixed strategies of the “weak” players and these payoff functions are non-linear.

For the mixed strategies, Diekmann's (1993) analytical exploration implies that the defection probability of “strong” and “weak” players will be: 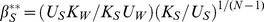 and 
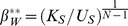
 respectively, indicating that the defection probability of “strong” players is greater than for “weak” players. However, this result is contradictory to the concept of Schelling (i.e. prominent solution) that the “strong” player produces the public goods [8], [38], [40]. This is also contradictory to empirical observations that queens (“strong” player) police eggs in some species of social insects, while workers (“weak” player) police eggs in other species [28], [29], rather than the “strong” and “weak” players all produce public goods with mixed strategies.

### Dynamics and Evolutionary Stability of the Asymmetric Volunteer's Dilemma Game

In the asymmetric volunteer's dilemma game, we imagine a multi-population setting for the purpose of extending the static N-player game to an evolutionary game. This means that players in the model described previously (section 2.1), are replaced by populations in this model, as it has been done previously [30], [41]. We present an evolutionary game that describes how the frequencies of strategies within a population change in time according to the success of different strategists [42]–[44], and explore which equilibrium will survive over evolutionary time.

Here, we study a special case of the asymmetric volunteer's dilemma game with one “strong” population and *N-1* “weak” populations. Each population simultaneously decides whether to “cooperate” or “defect”. In addition, we assume that *N-1* “weak” populations have an equal degree of weakness (i.e. *N-1* “weak” populations gain equal payoffs). All “weak” populations are monomorphic in which these players adopt the same strategy in the system [39].

Combining the assumptions of the asymmetric volunteer's dilemma game model with the rule of replicator dynamics—that the per capita rate of growth is given by the difference between the payoff for the strategy type and the average payoff in the population [41], [45]–[47] - we can establish the replicator dynamics of the asymmetric volunteer's dilemma game as follows:
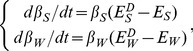
(2.4a)where 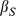 and 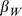 are the defection probabilities of the “strong” population/player and the “weak” populations/players respectively, and 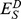/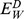 and 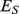/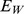 are respectively the payoff of the defection and the average payoffs of “strong/weak” populations/players, which are the same as described previously in section 2.1.

Substituted Equation 2.2 and 2.3 into Equation (2.4a), we can obtain
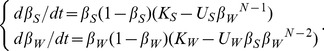
(2.4b)where 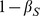 and 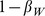 are the cooperation probabilities of the “strong” population/player and “weak” populations/players respectively.

Let
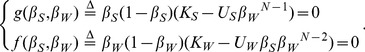
(2.5)


From the equations (2.5), we can obtain six equilibrium points of the system (2.4) (see Appendix) and denote them as: 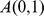, 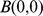, 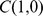, 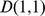, 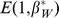, 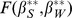, where elements of vector 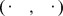 are respectively the frequencies of defection of “strong” and “weak” populations/players and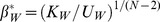, 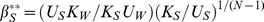, 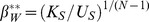.

It is noteworthy that the equilibrium point 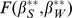 corresponds to the mixed strategy equilibrium of Diekmann's (1993) model, and that 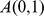, 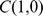 and 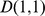 correspond to the pure strategy profiles of the replicator dynamics of the asymmetric volunteer's dilemma game (2.4). The equilibrium point 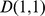 means all populations/players adopt the “free-rider” strategy. At the equilibrium point 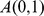, the “weak” population/player will adopt the “free-rider” strategy, but at the equilibrium point 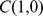, “strong” populations/players will adopt the “free-rider” strategy, while the opponents adopt “volunteer” at these two equilibriums points. The equilibrium point 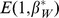 predicts that “weak” populations/players produce the public good while the “strong” population/player adopts the “free-rider” strategy. This equilibrium 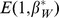 does not appear in Diekmann's (1993) results.

Clearly, the replicator dynamics of the asymmetric volunteer's dilemma game (2.4) is a nonlinear system, and we could therefore study the local asymptotic stability of equilibrium points by linearization. Therefore, the linearization of the replicator dynamics (2.4) at an equilibrium point 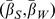 is
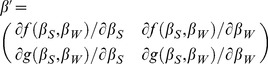
(2.6)Where 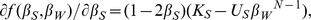 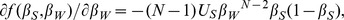 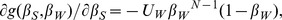 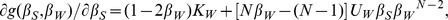 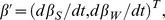 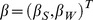 (here 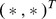 is the transpose of vector 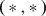), and 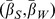
represents the equilibrium point, where 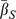 and 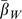 are respectively the frequencies of defection of “strong” and “weak” populations/players.

**Figure 1:**
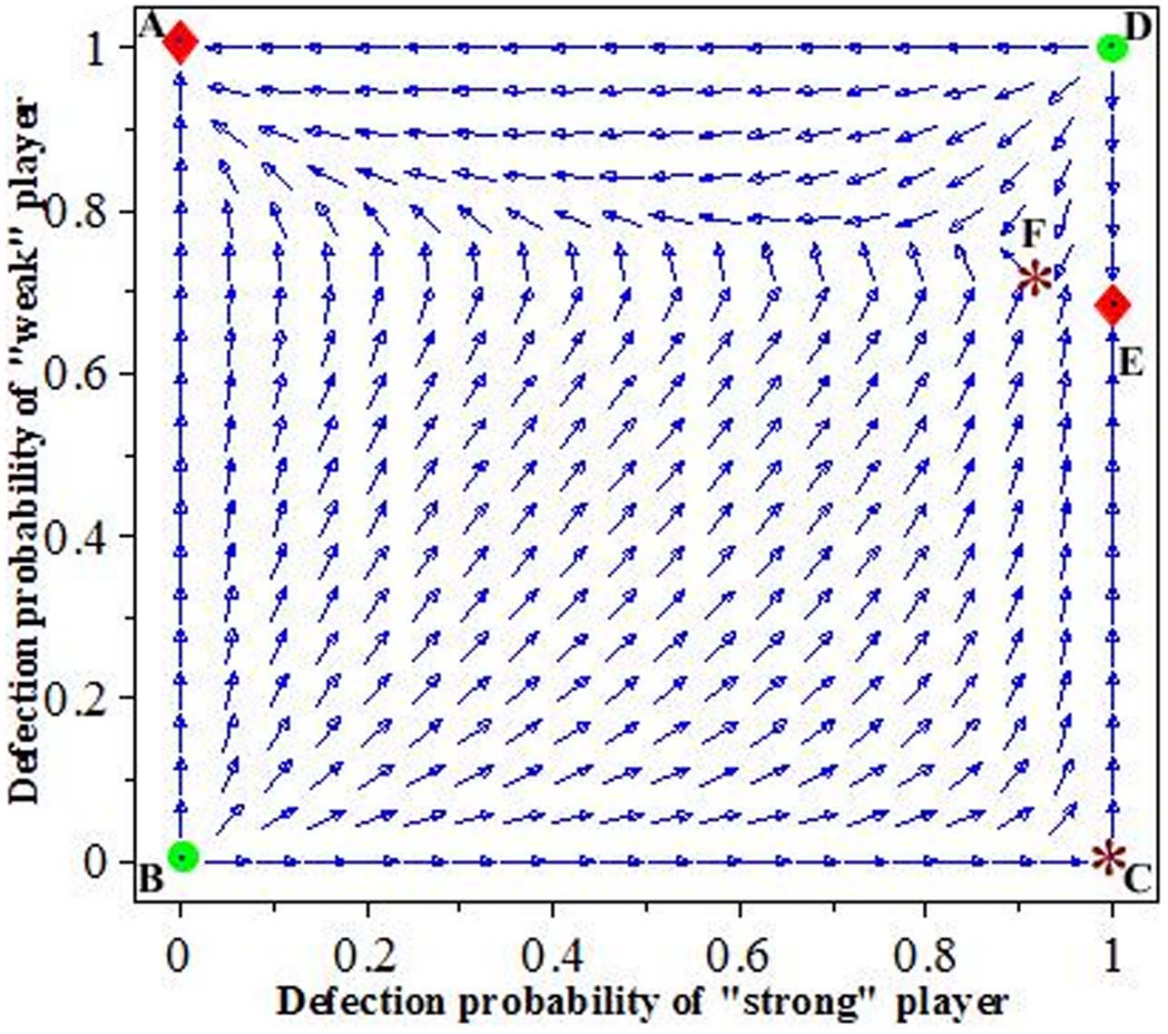
The slope field for replicator dynamics of the asymmetric volunteer's dilemma. Sink points 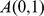 and 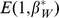 are the stable equilibrium solutions (red diamonds), and source points *B*, *D* (green circles) and saddle points *C*, *F* (purple red stars) are unstable equilibrium solutions. The parameters are fixed at 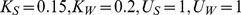 and 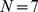.

**Figure 2:**
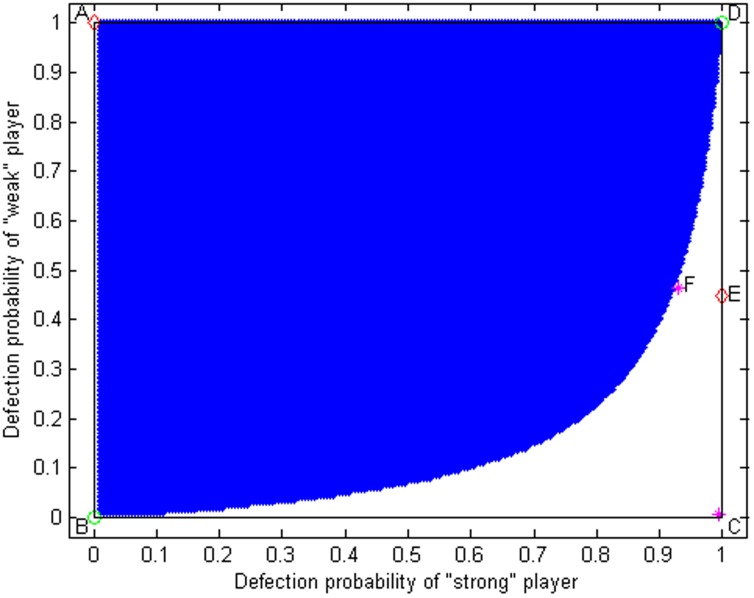
The basin of attraction of the local stable equilibrium points A (“shadow region”) and E (“blank region”). The parameters are fixed at 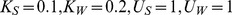 and 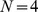.

**Figure 3:**
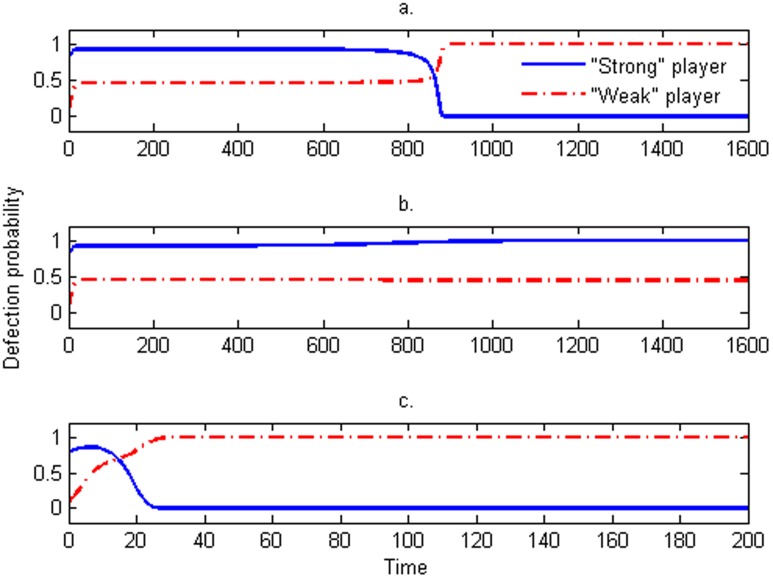
The numerical solution of the replicator dynamics for different initial values. The initial values 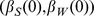 are 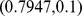, 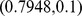 and 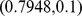 in (a), (b) and (c) respectively, where 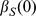 and 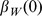 are the initial values of the defection probability of the “strong” player and the “weak” co-players respectively. The cost 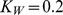 in (a) and (b) while 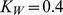 in (c). The other parameters are fixed at 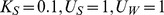 and 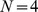.

By analyzing the property of the matrices' eigenvalues, produced by the linearization of the replicator dynamics at these six equilibrium points (see Appendix S1, Figure 1 and Figure 2), we find that 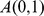 and 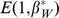 are sinks (stable), 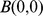 and 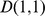 are sources (unstable), while 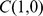 and 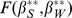 are saddles (unstable) [41], [48]. It is also noteworthy that the stability of equilibrium point 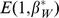 depends on the special condition, that is, the equilibrium point 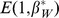 is only stable if and only if the cost-benefit ratio of the “strong/weak” player and the group size satisfy inequality 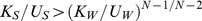 (see Figure 3 and Appendix S1). Furthermore, the rest point 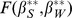 exists in the interior of the unit square if and only if this same inequality 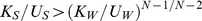 holds and the group size N is not too large (i.e. 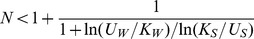). Thus, the replicator dynamics of the game are illustrated in two evolutionary stability points 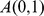 and 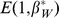 (Figure 1).

These two stable points 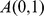 and 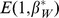 are local stability (see Appendix S1), which each have their own basins of attraction. Obtaining the basins of attraction of local stable equilibrium points has proven extremely useful for analyzing the behaviors of individuals, but they are difficult to obtain in nonlinear systems [48]–[50]. Using numerical simulations, we are able to obtain the properties of the basins of attraction in the replicator dynamics of the asymmetric volunteer's dilemma game. The “shadow region” and “blank region” (here, the boundary between shadow and blank regions go through the point F) are the basins of attraction of equilibrium points 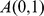 and 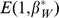 (Figure 2); in effect, the solution trajectories will converge to the point 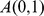 if the initial values of the system fall in the “shadow region,” while they converge to the point 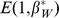 if the initial values of the system fall in the “blank region” (see Figures 2 and Figures 3).

## Results and Discussion

In the symmetric equilibrium of the volunteers' dilemma with symmetric costs, each player has an equal probability of cooperation [30], [41]. However, in real social dilemmas, costs may be asymmetric, and the payoffs might therefore be unequal [24], [31], [35], [51]. The model we have developed is the volunteers' dilemma with one “strong” population/player and *N-1* “weak” populations/players. Using evolutionarily game theory, our results show that the mixed strategy used by Diekmann (1993) is not evolutionary stable, that is, the paradox that the defection probability of the “strong” population/player is greater than the “weak” populations/players disappears in our evolutionary analysis. Similar to our demonstration here, Selten (1980) previously demonstrated that a mixed strategy will never achieve an evolutionarily stable strategy in asymmetric games [37], [41], [52]. Furthermore, the model we present here shows that two evolutionarily stable strategies might exists in an asymmetric cooperation system, and whether the “strong” population/player or “weak” populations/players produce the public good will depend on the initial condition of the system (Figures 2 and Figures 3).

From the analysis presented in section 2.2, the “strong” population/player will produce the public good if the initial condition of the systems fall in the basin of attraction of local stable equilibrium points 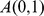 (the “shadow region” in Figure 2), and it yields a pareto-efficient equilibrium. Conversely, if the initial conditions of the system fall in the basin of attraction of local stable equilibrium points 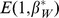 (the “blank region” in Figure 2), the public good is produced by the “weak” populations/players. These simulations also show that the evolutionary stability of this system is sensitive to the initial conditions (Figure 3a,b).

In our model, the public goods of the asymmetric system is produced by the “strong” population/player under some initial conditions, and this result is similar to previous models [37], [52]. Moreover, the model we developed also predicts that “weak” populations/players might also produce the public good under other initial conditions. The different initial conditions correspond with the initial states of individual strategies in our model, while the initial states of individual strategies could be defined as the phenotype or initial strategy of the individuals [53]. The phenotype or initial strategy of individuals might be inherited from parents [30], [54]. For instance, the social rank of the offspring of the spotted hyena depends on the rank of their mothers [55]–[58]. The phenotype or initial strategy of individuals (i.e., initial state of individual strategy) might also greatly be affected by the juvenile environment, not just determined by genetics (i.e., inheritance from its parents) [59]. Synthesizing the above-mentioned analysis, varying initial states might stem from the differences of both inheritance and habitat.

It is necessary to point out that the ESS 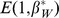 is a boundary strategy equilibrium, which implies that the “strong” population/player always defects and the *N-1* “weak” populations/players defect with a probability 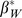. When the “weak” populations/players produce the public good, we see that the cooperation probability of “weak” populations/players decreases with increasing group size (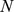) or decreasing cost-benefit ratio (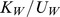), since the probability of volunteering for “weak” populations/players is 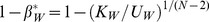. In the ESS 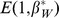, the probability of the production of the public good is 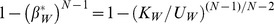, whose limit value 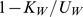 is lower than the probability of the production of the public good (*1*) by the “strong” population/player in the ESS 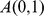.

## Supporting Information

Appendix S1 **Supporting information for “The equilibrium points and Local stability analysis”.**
(DOC)
