# Supplementary material for: Evolutionary Stability in the Asymmetric Volunteer's Dilemma
Source: PLoS One. 2014 Aug 11;9(8):e103931. doi: 10.1371/journal.pone.0103931 (PMC4128801; doi:10.1371/journal.pone.0103931)
Supplement: Appendix S1 — Supporting information for “The equilibrium points and Local stability analysis”. (DOC) [file pone.0103931.s001.doc]

**Evolutionary Stability in the Asymmetric Volunteer’s Dilemma**

***Supporting Information***

Jun-Zhou He, Rui-Wu Wang and Yao-Tang Li

**Appendix S1: The equilibrium points and Local stability analysis**

From the definition of equilibrium point of nonlinear systems, we know that the equilibrium points of the equation (2.3) in text are the solutions of the following equations

$\left\{\begin{matrix} \beta_{S}(1-\beta_{S})(K_{S}-U_{S}\beta_{W}^{N-1})=0\begin{matrix} & \end{matrix} \\ \beta_{W}(1-\beta_{W})(K_{W}-U_{W}\beta_{S}\beta_{W}^{N-2})=0 \end{matrix}\right.$. (1)

Now, we solve the equation (1). Solving$\beta_{S}(1-\beta_{S})(K_{S}-U_{S}\beta_{W}^{N-1})=0$, we get

$\beta_{S}=0,\begin{matrix} \end{matrix}\beta_{S}=1,\begin{matrix} \end{matrix}\beta_{W}=\left(K_{S}/U_{S}\right)^{1/(N-1)}$.

Solving $\beta_{W}(1-\beta_{W})(K_{W}-U_{W}\beta_{S}\beta_{W}^{N-2})=0$, we get $\beta_{W}=0,\begin{matrix} \end{matrix}\beta_{W}=1$ and

$K_{W}-U_{W}\beta_{S}\beta_{W}^{N-2}=0$. (2)

Obviously, points$(\bar{\beta}_{S},\bar{\beta}_{W})=(0,1),\begin{matrix} \end{matrix}(0,0),\begin{matrix} \end{matrix}(1,0),\begin{matrix} \end{matrix}(1,1)$ are the solutions of the equations (1). We denote them as $A(0,1)$, $B(0,0)$, $C(1,0)$, $D(1,1)$, respectively, where $\bar{\beta}_{S}$and $\bar{\beta}_{W}$ are respectively the frequencies of defectors of “strong” and “weak” players.

Substituting $\beta_{S}=1$ in Equation (2) yields:

$\beta_{W}=\left(K_{W}/U_{W}\right)^{1/(N-2)}$，

that is, $\left(\bar{\beta}_{S},\bar{\beta}_{W}\right)=\left(1,\begin{matrix} \end{matrix}\left(K_{W}/U_{W}\right)^{1/(N-2)}\right)$ is also a solution of the equations (1), and we denote it as $E(1,\beta_{W}^{*})$，here $\beta_{W}^{*}=\left(K_{W}/U_{W}\right)^{1/(N-2)}$.

Substituting $\beta_{W}=\left(K_{S}/U_{S}\right)^{1/(N-1)}$in Equation (2), we obtain

$$\begin{matrix} \beta_{S}=\left(K_{W}/U_{W}\right)\left(K_{S}/U_{S}\right)^{(N-2)/(N-1)} \\ \begin{matrix} \end{matrix}=\left(K_{W}/U_{W}\right)\left(K_{S}/U_{S}\right)^{(N-1-1)/(N-1)} \\ \begin{matrix} \end{matrix}=\left(K_{W}/U_{W}\right)\left(K_{S}/U_{S}\right)\left(K_{S}/U_{S}\right)^{-1/(N-1)} \\ \begin{matrix} \end{matrix}=\left(U_{S}K_{W}/K_{S}U_{W}\right)\left(K_{S}/U_{S}\right)^{1/(N-1)}. \end{matrix}$$

So $\left(\bar{\beta}_{S},\bar{\beta}_{W}\right)=\left(\left(U_{S}K_{W}/K_{S}U_{W}\right)\left(K_{S}/U_{S}\right)^{1/(N-1)},\begin{matrix} \end{matrix}\left(K_{S}/U_{S}\right)^{1/(N-1)}\right)$ is also a solution of the equations (1), and we denote it as $F(\beta_{S}^{**},\beta_{W}^{**})$，here $\beta_{S}^{**}=\left(U_{S}K_{W}/K_{S}U_{W}\right)\left(K_{S}/U_{S}\right)^{1/(N-1)}$ and $\beta_{W}^{**}=\left(K_{S}/U_{S}\right)^{1/(N-1)}$.

From above discussion, we obtain six equilibrium points of the nonlinear system (2.3): $A(0,1)$, $B(0,0)$, $C(1,0)$, $D(1,1)$, $E(1,\beta_{W}^{*})$, $F(\beta_{S}^{**},\beta_{W}^{**})$,

where$\beta_{W}^{*}=\left(K_{W}/U_{W}\right)^{1/(N-2)}$, $\beta_{S}^{**}=\left(U_{S}K_{W}/K_{S}U_{W}\right)\left(K_{S}/U_{S}\right)^{1/(N-1)}$ , $\beta_{W}^{**}=\left(K_{S}/U_{S}\right)^{1/(N-1)}$.

The following is the local stability analysis of the system of volunteer dilemma game. In text, the linearization of the replicator dynamics (2.3) at an equilibrium point $(\bar{\beta}_{S},\bar{\beta}_{W})$ is

$\beta'=\left.\left(\begin{matrix} \partial f(\beta_{S},\beta_{W})/\partial\beta_{S} & \partial f(\beta_{S},\beta_{W})/\partial\beta_{W} \\ \partial g(\beta_{S},\beta_{W})/\partial\beta_{S} & \partial g(\beta_{S},\beta_{W})/\partial\beta_{S} \end{matrix}\right)\right|_{(\beta_{S},\beta_{W})=(\bar{\beta}_{S},\bar{\beta}_{W})}\beta$. (3)

For convenience, let

$J(\bar{\beta}_{S},\bar{\beta}_{W})=\left.\left(\begin{matrix} \partial f(\beta_{S},\beta_{W})/\partial\beta_{S} & \partial f(\beta_{S},\beta_{W})/\partial\beta_{W} \\ \partial g(\beta_{S},\beta_{W})/\partial\beta_{S} & \partial g(\beta_{S},\beta_{W})/\partial\beta_{S} \end{matrix}\right)\right|_{(\beta_{S},\beta_{W})=(\bar{\beta}_{S},\bar{\beta}_{W})}$， (4)

where

$\partial f(\beta_{S},\beta_{W})/\partial\beta_{S}=(1-2\beta_{S})(K_{S}-U_{S}\beta_{W}^{N-1})$,

$\partial f(\beta_{S},\beta_{W})/\partial\beta_{W}=-(N-1)U_{S}\beta_{W}^{N-2}\beta_{S}(1-\beta_{S})$,

$\partial g(\beta_{S},\beta_{W})/\partial\beta_{S}=-U_{W}\beta_{W}^{N-1}(1-\beta_{W})$,

$\partial g(\beta_{S},\beta_{W})/\partial\beta_{S}=(1-2\beta_{W})K_{W}+[N\beta_{W}-(N-1)]U_{W}\beta_{S}\beta_{W}^{N-2}$,

and $(\bar{\beta}_{S},\bar{\beta}_{W})$represents the equilibrium points of the nonlinear system (2.3), $\bar{\beta}_{S}$and $\bar{\beta}_{W}$ are respectively the frequencies of defection of “strong” and “weak” players.

From (4), we obtain the Jacobi matrices of the linear system (3) of the nonlinear system (2.3) at these six equilibrium points:

$J(0,0)=\left(\begin{matrix} K_{S} & 0 \\ 0 & K_{W} \end{matrix}\right)$, $J(0,1)=\left(\begin{matrix} K_{S}-U_{S} & 0 \\ 0 & -K_{W} \end{matrix}\right)$, $J(1,0)=\left(\begin{matrix} -K_{S} & 0 \\ 0 & K_{W} \end{matrix}\right)$,

$J(1,1)=\left(\begin{matrix} U_{S}-K_{S} & 0 \\ 0 & U_{W}-K_{W} \end{matrix}\right)$, $J(1,\beta_{W}^{*})=\left(\begin{matrix} -[K_{S}-U_{S}(\beta_{W}^{*})^{N-1}] & 0 \\ 0 & -(N-2)K_{W}(1-\beta_{W}^{*}) \end{matrix}\right)$

$J(\beta_{S}^{**},\beta_{W}^{**})=\left(\begin{matrix} 0 & -(N-1)U_{S}\beta_{S}^{**}(1-\beta_{S}^{**})(\beta_{W}^{**})^{N-2} \\ -U_{W}(1-\beta_{W}^{**})(\beta_{W}^{**})^{N-1} & -(N-2)U_{W}\beta_{S}^{**}(1-\beta_{W}^{**})(\beta_{W}^{**})^{N-2} \end{matrix}\right)$.

The local stability of the nonlinear system (2.3) at equilibrium points is determined by the eigenvalues of the Jacobi matrices. Now, we analyze character of the eigenvalues of above six matrices.

Since $0<K_{S}<K_{W}<U_{W}\leq U_{S}$and $J(0,0)$,$J(1,1)$,$J(0,1)$and $J(1,0)$are diagonal matrices, we can easily get that the eigenvalues of $J(0,0)$ and $J(1,1)$ are all positive, and the eigenvalues of $J(0,1)$ are negative. On thus condition, one of the eigenvalues of $J(1,0)$ is positive and another is negative.

For $J(1,\beta_{W}^{*})$, we obtain easily that its eigenvalues are$-(N-2)K_{W}(1-\beta_{W}^{*})$and $-[K_{S}-U_{S}(\beta_{W}^{*})^{N-1}]$. Obviously, $-(N-2)K_{W}(1-\beta_{W}^{*})<0$. In addition, from $0<K_{S}/U_{S}<K_{W}/U_{W}<1$, we know that yield $-[K_{S}-U_{S}(\beta_{W}^{*})^{N-1}]<0$ (here $\beta_{W}^{*}=\left(K_{W}/U_{W}\right)^{1/N-2}$) demand $K_{S}/U_{S}>\left(K_{W}/U_{W}\right)^{N-1/N-2}$. So another eigenvalue is negative if and only if $K_{S}/U_{S}>\left(K_{W}/U_{W}\right)^{N-1/N-2}$, that is, the eigenvalues of $J(1,\beta_{W}^{*})$ are all negative under the special condition $\left(K_{S}/U_{S}\right)^{N-2}>\left(K_{W}/U_{W}\right)^{N-1}$.

As to $J(\beta_{S}^{**},\beta_{W}^{**})$, its characteristic equation is

$\lambda^{2}-a_{22}\lambda-a_{12}a_{21}=0$. (8)

Solving Equation (8), we get

$\lambda=\frac{a_{22}\pm\sqrt{a_{22}^{2}+4a_{12}a_{21}}}{2}$，

where

$a_{12}=-(N-1)U_{S}\beta_{S}^{**}(1-\beta_{S}^{**})(\beta_{W}^{**})^{N-2}$，

$a_{21}=-U_{W}(1-\beta_{W}^{**})(\beta_{W}^{**})^{N-1}$，

$a_{22}=-(N-2)U_{W}\beta_{S}^{**}(1-\beta_{W}^{**})(\beta_{W}^{**})^{N-2}$.

Obviously,$a_{12}<0,\begin{matrix} \end{matrix}a_{21}<0$ and $a_{22}<0$, then it is easy to obtain $\left|a_{22}\right|<\sqrt{a_{22}^{2}+4a_{12}a_{21}}$. Hence, one of the eigenvalues of $J(\beta_{S}^{**},\beta_{W}^{**})$ is a positive real number and another is a negative.

From above analysis of the eigenvalues and the stability theory of differential equations (Hofbauer and Sigmund 1998; Hirsch et al. 2004), we obtain that $A(0,1)$ and $E(1,\beta_{W}^{*})$ are sinks (stable); and that$B(0,0)$ and $D(1,1)$ are sources (unstable); $C(1,0)$ and $F(\beta_{S}^{**},\beta_{W}^{**})$ are saddles (unstable).

**References**

Hirsch, M. W., Smale, S., Devaney, R. L., 2004. *Differential Equations, Dynamical Systems, and an Introduction to Chaos.* Calif. Acad. Press.

Hofbauer, J., Sigmund, K., 1998. *Evolutionary Games and Population Dynamics.* Cambridge Uni. Press.
